# Supplementary material for: Cancer of unknown primary stem-like cells model multi-organ metastasis and unveil liability to MEK inhibition
Source: Nat Commun. 2021 May 3;12:2498. doi: 10.1038/s41467-021-22643-w (PMC8093243; doi:10.1038/s41467-021-22643-w)
Supplement: Supplementary file 1 — Supplementary Information [file 41467_2021_22643_MOESM1_ESM.pdf]

**Supplementary Information**

**Cancer of Unknown Primary stem-like cells model multi-organ metastasis and unveil liability to MEK inhibition**

Federica Verginelli, Alberto Pisacane, Gennaro Gambardella, Antonio D'Ambrosio, Ermes Candiello, Marco Ferrio, Mara Panero, Laura Casorzo, Silvia Benvenuti, Eliano Cascardi, Rebecca Senetta, Elena Geuna, Andrea Ballabio, Filippo Montemurro, Anna Sapino, Paolo M. Comoglio, and Carla Boccaccio

**Inventory of Supplementary Information**

**1.**

**Supplementary Figures**

**Supplementary Figure 1**

**Supplementary Figure 2**

**Supplementary Figure 3**

**Supplementary Figure 4**

**2.**

**Supplementary  Tables**

**Supplementary Table 1**

**Supplementary Table 2**

**Supplementary Table 3**

**3.**

**Supplementary Notes**

**4.**

**Supplementary Methods**

**5.**

**Supplementary References**

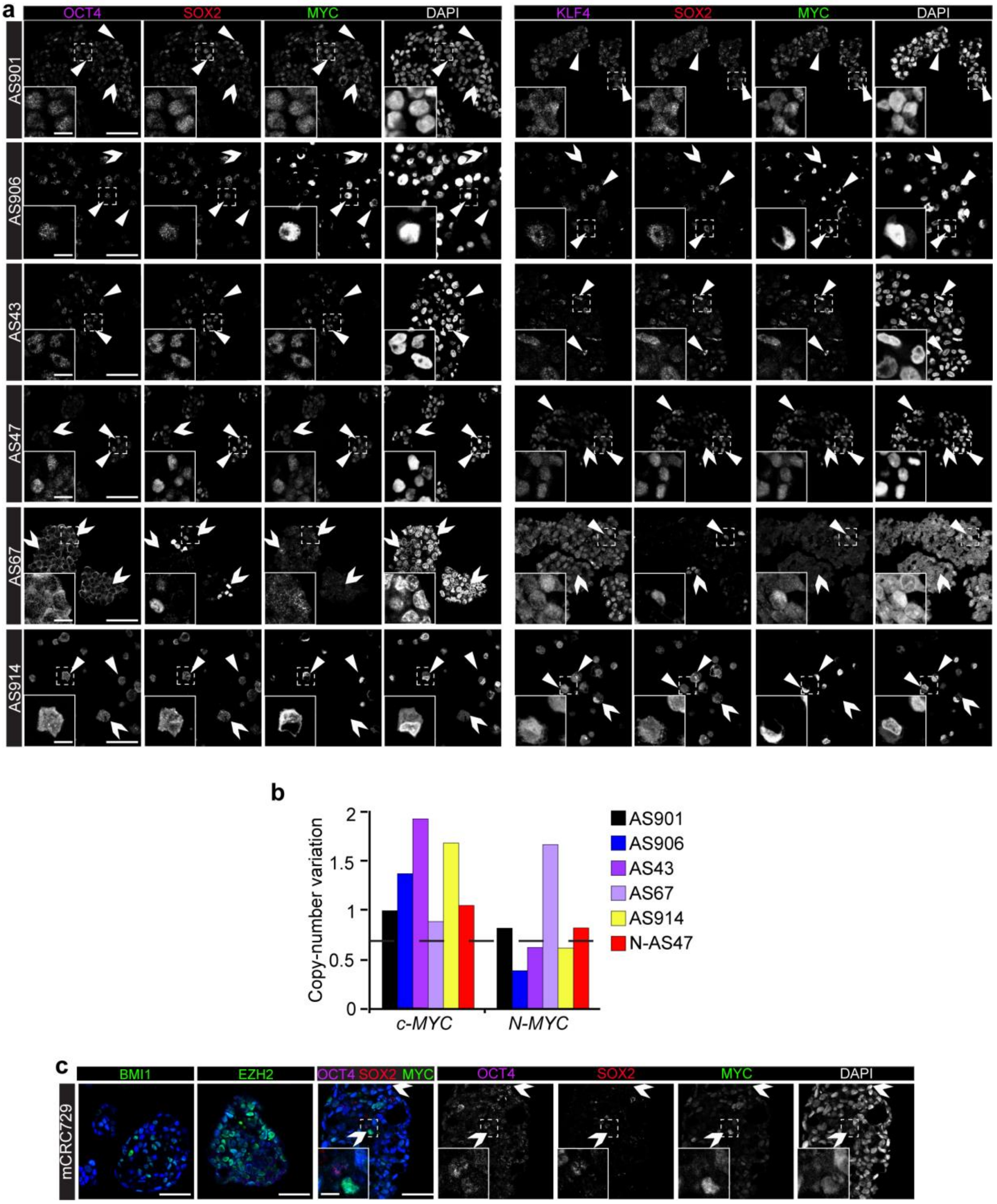
a
OCT4
SOX2
MYC
DAPI
KLF4
SOX2
MYC
DAPI
AS901
AS906
AS43
AS47
AS67
AS914
b
Copy-number variation
2
1.5
1
0.5
0
c-MYC
N-MYC
AS901
AS906
AS43
AS67
AS914
N-AS47
c
BMI1
EZH2
OCT4 SOX2 MYC
OCT4
SOX2
MYC
DAPI
mCRC729

d

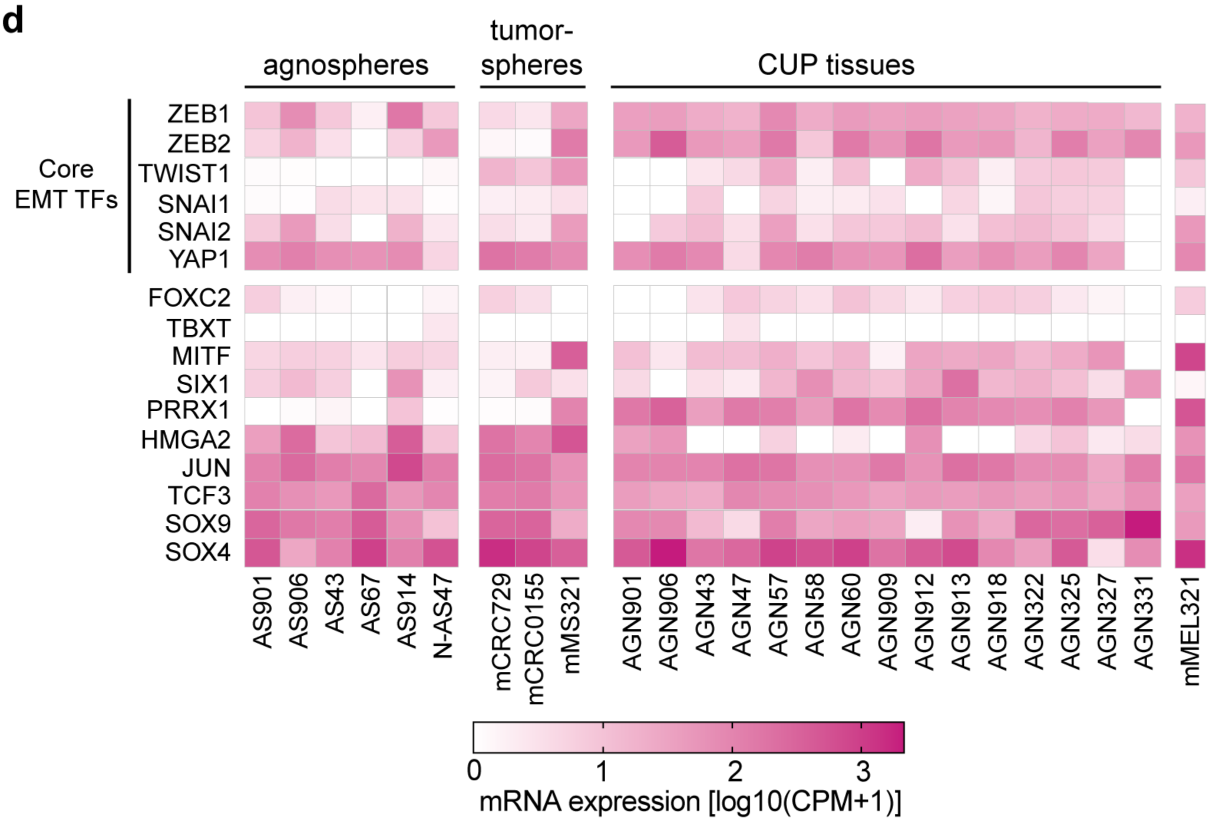

agnospheres
tumor-spheres
CUP tissues
Core EMT TFs
ZEB1
ZEB2
TWIST1
SNAI1
SNAI2
YAP1
FOXC2
TBXT
MITF
SIX1
PRRX1
HMGA2
JUN
TCF3
SOX9
SOX4
AS901
AS906
AS43
AS67
AS914
N-AS47
mCRC729
mCRC0155
mMS321
AGN901
AGN906
AGN43
AGN47
AGN57
AGN58
AGN60
AGN909
AGN912
AGN913
AGN918
AGN322
AGN325
AGN327
AGN331
mMEL321
0
1
2
3
mRNA expression [log10(CPM+1)]


e

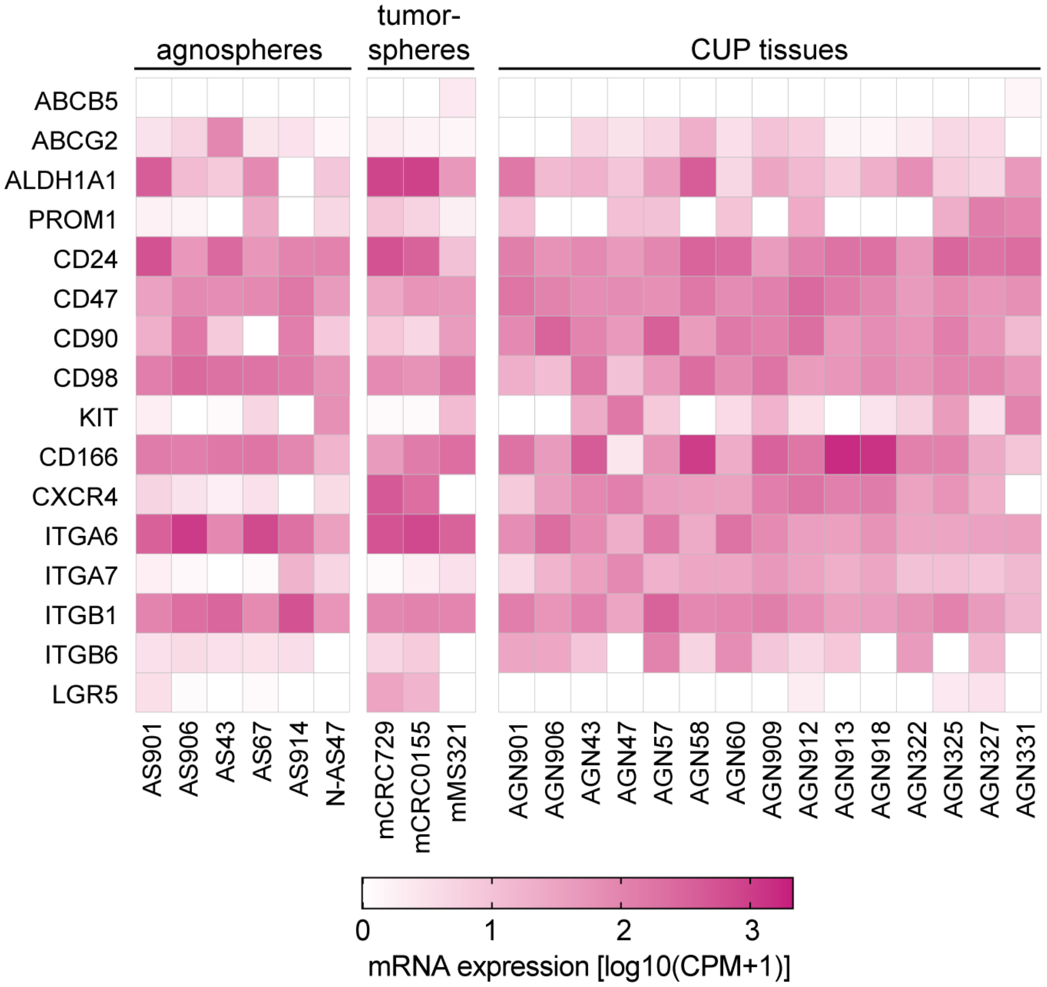

agnospheres
tumor-spheres
CUP tissues
ABCB5
ABCG2
ALDH1A1
PROM1
CD24
CD47
CD90
CD98
KIT
CD166
CXCR4
ITGA6
ITGA7
ITGB1
ITGB6
LGR5
AS901
AS906
AS43
AS67
AS914
N-AS47
mCRC729
mCRC0155
mMS321
AGN901
AGN906
AGN43
AGN47
AGN57
AGN58
AGN60
AGN909
AGN912
AGN913
AGN918
AGN322
AGN325
AGN327
AGN331
0
1
2
3
mRNA expression [log10(CPM+1)]

**Supplementary Figure 1.** Agnospheres and CUP tissues are enriched in stem-like cells. **a,** Images of single split channels of triple immunofluorescence stainings shown in Fig. 2b. Arrowheads point to examples of cells with overlapping marker expression, open arrows point to examples of cells with single marker expression. A representative experiment is shown (n=3 independent stainings of agnospheres with similar results were obtained). Scale bar, 50 μm. For each channel a magnification of the dotted area is shown in the inset. Scale bar, 10μm. **b**, c-*MYC* and N-*MYC* copy-number variations measured by qPCR in gDNA from agnospheres. Value=1 indicates biallelic content. Values<0.7 (dotted line) indicate allelic loss. Gene amplification is defined by copy number variation>6 (n=2 independent experiments with similar results were obtained). **c**, Immunofluorescence staining of colosphere mCRC729 for the indicated markers. A representative experiment is shown (n=3 independent stainings of colospheres with similar results were obtained). Scale bar, 50 μm. Single split channels are shown for triple labelling. Inset: magnification of the dotted area. Scale bar, 10μm. **d-e**, Heatmaps showing gene expression levels, analyzed by 3' UTR-seq, of transcription factors (TFs) associated with Epithelial-Mesenchymal Transition (EMT) (**d**), and functional and cell-surface markers known to be associated with stem-like cells in carcinomas of known origin (**e**). mMEL321: melanoma metastasis tissue corresponding to melanosphere mMS321.

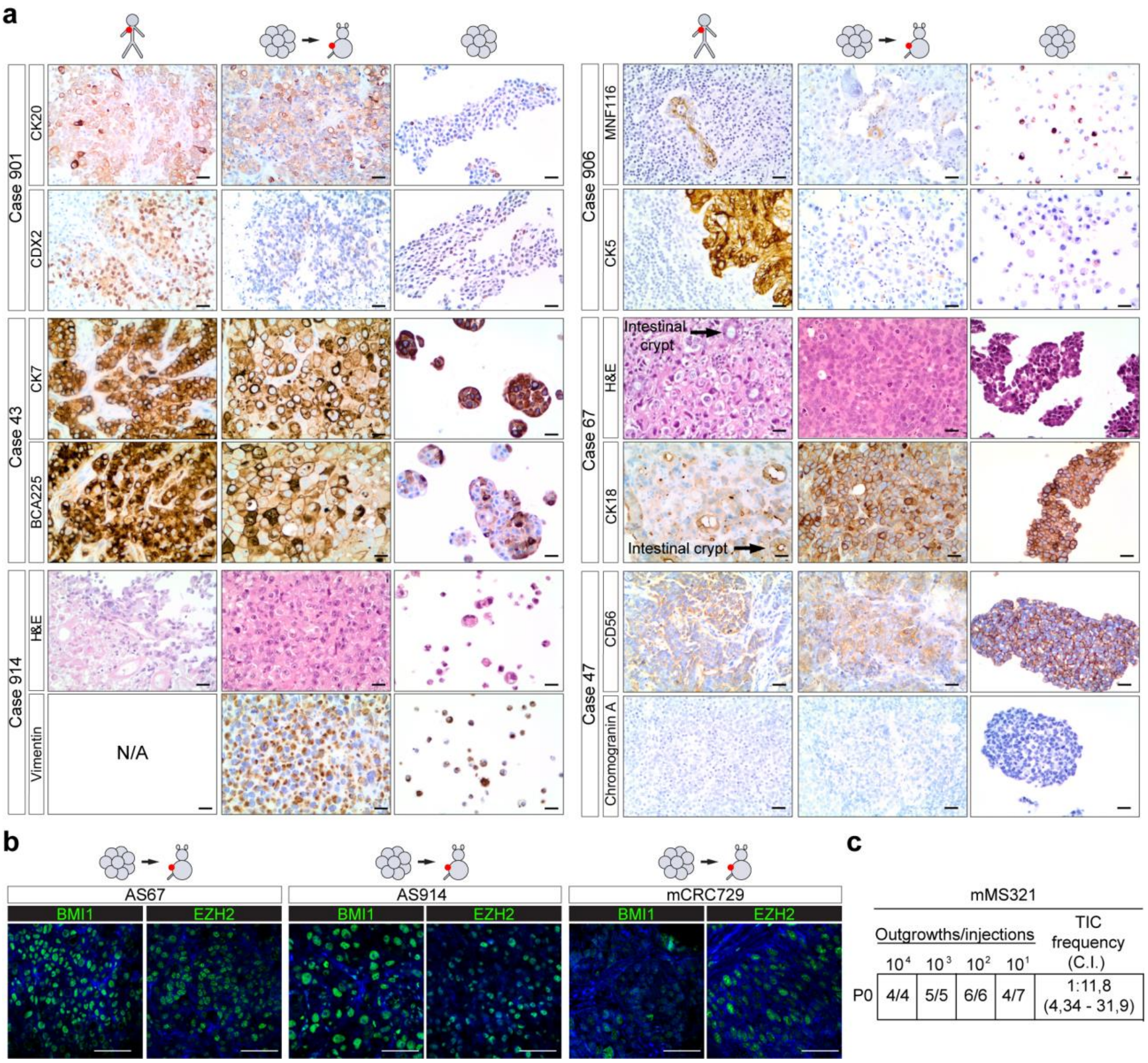


**Supplementary Figure 2.** Agnospheres generate phenocopies of the original tumors. **a**, Histopathological features (H&E) and immunohistochemistry with antibodies used for CUP diagnosis, in: patients' metastases, tumors formed at the subcutaneous injection site (IS-tumor) and agnospheres. CDX2 and CK5 are expressed only in focal areas of original tumors AGN901 and AGN906, respectively (see Supplementary Table 1). A representative image of IS-tumor is shown (n=3 independent stainings, for each marker similar results were obtained). The patient tumor tissue of case 67 includes intestinal crypts (arrows) as it was excised from a mass infiltrating the colonic wall, carefully excluded to be a primary site. Scale bar, 50 $\mu$m. **b**, Immunofluorescent staining of stem

cell markers in tumors formed at the subcutaneous injection site of agnospheres AS67 and AS914 and metastatic colosphere mCRC729. A representative image of IS-tumor is shown (n=3 independent stainings, for each marker similar results were obtained). Scale bar, 50 μm. BCA225: breast cancer antigen 225; CDX2: caudal type homeobox 2; CK: cytokeratin; H&E: hematoxylin and eosin; N/A: not available. **c,** *In vivo* limiting dilution assay of melanosphere mMS321. The indicated numbers of melanosphere cells ($10^4$-$10^1$) were transplanted *subcutis* into immunocompromised mice (P0). TIC: Tumor-initiating cell frequency. C.I.: confidence interval.

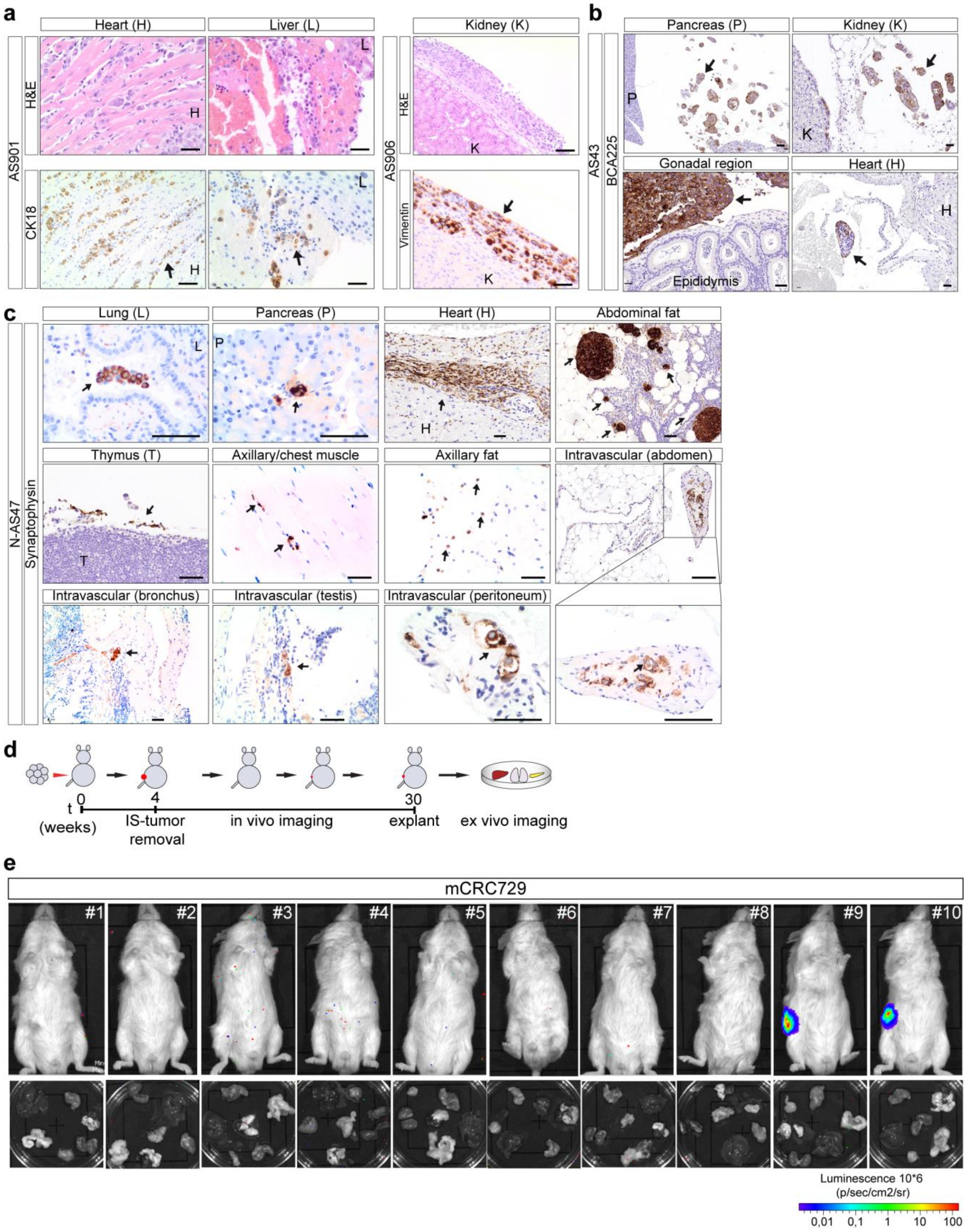


**Supplementary Figure 3.** Subcutaneously transplanted agnospheres reproduce the early and multi-organ metastatic pattern of CUP patients while spheres from metastatic

colorectal cancer do not metastasize. **a-c**, Histopathology (H&E staining) and immunohistochemistry of metastases in the indicated organs from spheropatients, performed with human-specific antibodies against markers ubiquitously expressed in the original CUPs. Representative images are shown for each organ. **a**, AS901 spheropatient, immunostaining for Cytokeratin 18 (CK18); AS906 spheropatient, immunostaining for Vimentin. Scale bar, 50 μm. **b**, AS43 spheropatient, immunostaining for BCA225. Scale bar, 50 μm. **c**, N-AS47 spheropatient, immunostaining for Synaptophysin. Inset: image magnification is shown in the row below. Scale bar, 50 μm. **d**, Outline of longitudinal *in vivo* monitoring of spheropatients transplanted *subcutis* with luciferase-labelled colosphere mCRC729, followed by end-point *ex-vivo* organ imaging with IVIS. IS-tumor: injection site tumor. **e**, Representative *in vivo* images of mice transplanted with mCRC729 (n=14 mice) and ex-vivo images of different organs (bottom), taken 30 weeks after transplantation, showing absence of bioluminescent signals suggestive of metastases. Signals detected in mice #9 and #10 (top) correspond to local regrowth after IS-tumor removal.

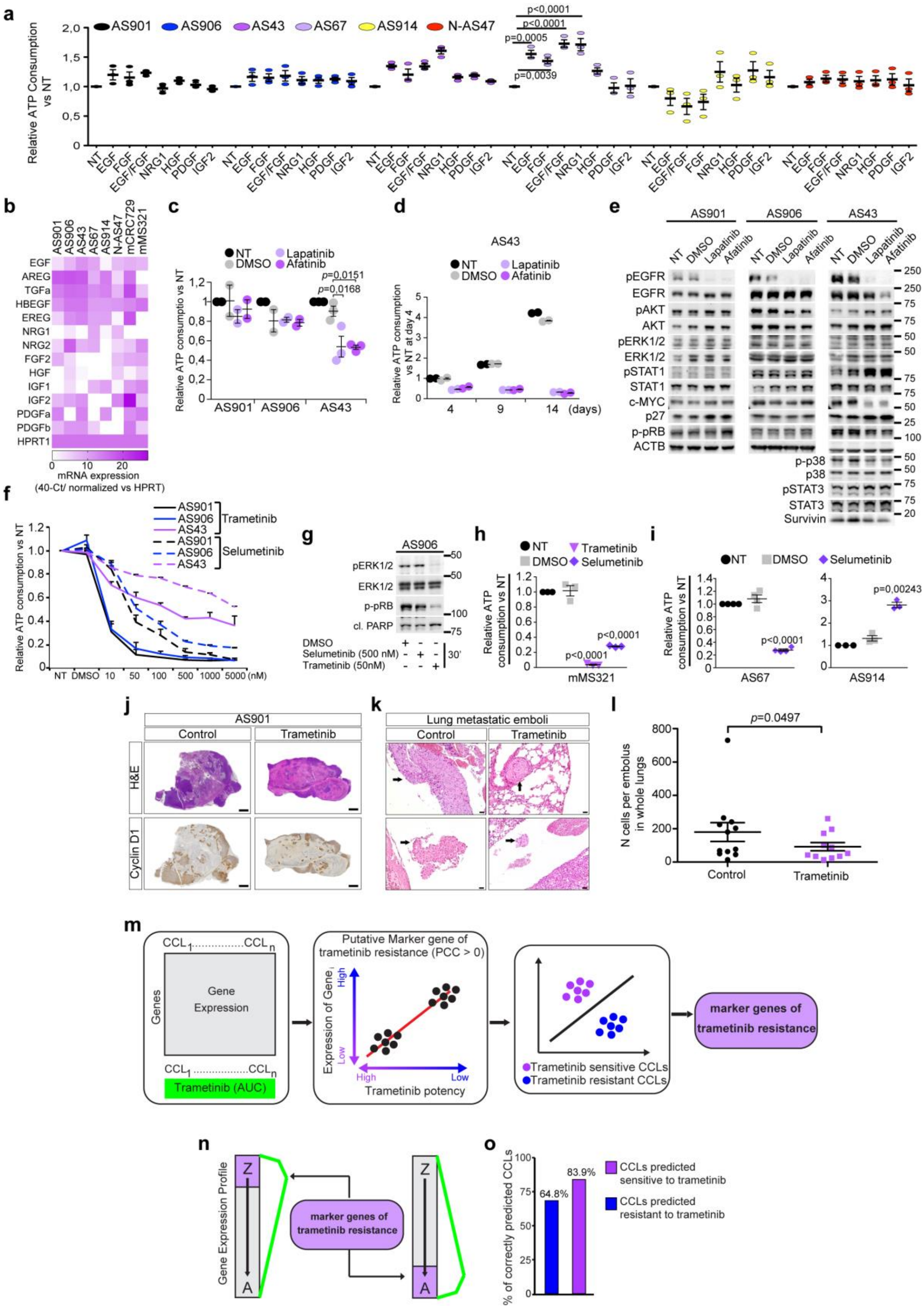


**Supplementary Figure 4.** Agnosphere response to exogenous growth factors and EGFR family or MEK inhibitors, and generation of a trametinib response signature. **a**,

Agnosphere viability evaluated 4 days after supply of the indicated growth factors through quantification of relative ATP consumption normalized vs. untreated condition (NT) (n=3 independent experiments, mean±SEM, ANOVA, Bonferroni multicomparison test). **b**, Heatmaps showing expression levels of endogenous mRNAs of the indicated growth factors in agnospheres and tumorspheres (mCRC729 and mMS321), measured by qRT-PCR and expressed as 40-Ct. HPRT was used as normalizer (n=2 independent experiments, similar results were obtained). **c,** Agnosphere viability evaluated 4 days after treatment with the indicated EGFR inhibitors, lapatinib (0.5 μm) or afatinib (0.5 μm), or vehicle (DMSO), through quantification of relative ATP consumption normalized vs. untreated condition (NT) (AS901 n=2, AS906 n=2, AS43 n=3 independent experiments, mean±SEM, ANOVA, Bonferroni multicomparison test: Lapatinib vs. DMSO and Afatinib vs. DMSO). **d**, AS43 viability evaluated at the indicated time-points after treatment with the indicated EGFR inhibitors or vehicle (DMSO) through quantification of relative ATP consumption normalized vs. untreated condition (NT) at day 4 (n=2, independent experiments, mean). **e,** Representative western blot analysis of agnospheres treated 4 days with the indicated inhibitors (n=2 independent experiments, similar results were obtained). pAKT: phospho-AKT; pERK1/2: phospho-ERK1/2; p-p38: phospho-p38-MAPK; pSTAT1: phospho-STAT1; pSTAT3: phospho-STAT3; p27: p27-KIP1; p-pRB: phospho-pRB. Actin B was used as loading control. **f**, Cell viability measured in agnospheres treated for 4 days with the indicated doses of trametinib, selumetinib or vehicle (DMSO) through quantification of relative ATP consumption normalized vs. untreated cells (NT) (trametinib: AS901 n=4, AS906 and AS43 n=3, and selumetinib: AS901 n=4, AS906 and AS43 n=5 independent experiments, mean±SEM). **g**, Representative western blot analysis of AS906 treated 30 min with selumetinib (500nM), trametinib (50nM) or vehicle (n=2 independent experiments, similar results were obtained); cl-PARP: cleaved PARP. **h-i,** Cell viability of melanosphere mMS321 (**h**) and agnospheres AS67 and AS914 (**i**), treated

with selumetinib (500 nM), or trametinib (50 nM), or vehicle (DMSO), measured through quantification of relative ATP consumption normalized vs. untreated condition (NT) at day 10 (mMS321 n=3, AS67 n=4, AS914 n=3 independent experiments, ANOVA, Bonferroni multicomparison test: selumetinib or trametinib vs DMSO). **j**, Representative hematoxylin and eosin (H&E) and immunohistochemical staining for Cyclin D1 of whole tumor sections derived from spheropatients transplanted with AS901 and treated with trametinib or control (n=3 mice). Scale bar, 2 mm. Quantification is shown in Fig. 5g (n=3 whole sections from independent tumors, mean±SEM, two-way ANOVA, Sidak's multicomparison test, ns, not significant). **k**, Lung sections from spheropatients transplanted with AS43, treated with control or trametinib and sacrificed at the experimental endpoint (tumor volume=1600 $mm^3$). Representative hematoxylin and eosin stainings. Arrows point to metastatic emboli. Scale bar 50μm. **l**, Quantification of metastatic emboli identified in whole lungs. After complete lung sectioning and staining, the total number of metastatic emboli was counted in each lung pair. In each embolus, the total number of cells was counted across sections spaced 20μm (1 out of 4 serial sections) to avoid duplicating counts of the same cell. The dot plot reports the number of cancer cells/embolus in control (12 emboli/1 mouse) or trametinib-treated mice (11 emboli/3 mice) (mean±SEM, Wilcoxon test, one-tail). **m-o**, Schematic representation of the pipeline used to generate the signature used to predict trametinib response. Briefly, expression of each gene is correlated with trametinib potency across 445 cancer cell lines (CCLs) downloaded from the Cancer Cell Line Encyclopedia. The top 1,000 positively correlated genes are used as input of a recursive feature elimination algorithm to select best predictive genes of trametinib response. **o**, Bar graph showing the accuracy of the signature in predicting resistance or sensitivity to trametinib in an independent dataset of 634 CCLs (GDSC1000).

## Supplementary Table 1. Clinical characteristics of patients

**Patients with Cancer of Unknown Primary**

| Case ID | Sex | Age at diagnosis (years) | OS (m) | Metastatic sites at progression | Histopathology | Immunohistochemical phenotype |
|---|---|---|---|---|---|---|
| AGN901* | M | 65 | 11 | Multiple lymph nodes (supra- and sub-diaphragmatic), liver | CUP, poorly differentiated adenocarcinoma | CK7+, CK20+, CDX2+ (focal), CK18+, TTF1- |
| AGN906* | F | 68 | 17 | Multiple lymph nodes (axilla, mediastinum), scalp with bone infiltration | CUP, poorly differentiated carcinoma with sarcomatoid features and focal squamous areas | CK7+, CK20-, CK5+ (focal), MNF116+, Vimentin+, p40-, S100- |
| AGN43* | M | 49 | 16 | Multiple lymph nodes, skin, muscles, bones, multiple visceral sites (intestine, peritoneum, lung, liver and kidney) | CUP, poorly differentiated adenocarcinoma | CK7+, CK20+/-, BCA225+, AE1/AE3+, TTF1-, Napsin A-, AR-, PSA-, ER-, PR-, CGDFP15-, CDX2- |
| AGN67* | F | 60 | 8 | Multiple lymph nodes (supra- and sub-diaphragmatic), lung, liver, adrenal gland, colon, bone | CUP, poorly differentiated adenocarcinoma | CK7+, CK20-, CK18+, p63+, Villin+, CDX2-, HepPar-1-, TTF1- |
| AGN914* | F | 46 | 5 | Multiple lymph nodes (supra-diaphragmatic), pectoral muscle, lung, brain, adrenal gland, gluteus muscle, soft tissues | CUP, poorly differentiated carcinoma with sarcomatoid features | CK7-, CK20-, AE1/AE3+, Vim+, TTF1-, ER-, PR-, HER2-, LCA-, S100-, Chromogranin A-, Myoglobin-, HMB45- |
| AGN47* | F | 43 | > 52 (Alive 09/2020) | Lymph nodes (groin and iliac vessels) | CUP, neuroendocrine carcinoma | CD56+, Synaptophysin+, Chromogranin A- |
| AGN57 | F | 50 | 7 | Multiple visceral sites (diaphragmatic and abdominal wall lesions) | CUP, adenocarcinoma with focal mucinous areas | CK7+, CK20+, CDX2+, WT1-, EP1- |
| AGN58 | M | 58 | 17 | Brain | CUP, poorly differentiated carcinoma | CK7+, CK20+, CK5+, p63+, EMA+, GATA3+, CDX2-, TTF1-WT1-, D2-40-, Thyroglobulin-, Vimentin- |
| AGN60 | M | 52 | 18 | Multiple lymph nodes (supra- and sub-diaphragmatic) | CUP, poorly differentiated carcinoma with squamous differentiation | CK7+, CK20-, CK5+, p63+, p40+, AE1/AE3+, CK34β12+, CD10+, CDX2-, TTF1-, PSA-, Inhibin A- |
| AGN909* | F | 49 | 19 | Multiple lymph nodes (supra- and sub-diaphragmatic), liver, bone, mammary, adrenal gland, skin, gluteus, ileo-psoas, abdominal wall | CUP, poorly differentiated carcinoma | CK7-, CK20-, CK19+, AE1/AE3+, CAM5.2+, Vimentin+, p63+/-, CDX2-, TTF1-, BCA225-, CGDFP15- |
| AGN912 | F | 69 | >68 (Alive 10/2020) | Multiple lymph nodes (supra- and sub-diaphragmatic) | CUP, poorly differentiated carcinoma | CK7+, CK20+, CK19+, WT1+, CA125+, p63+, BCA225+, PR+, ER-, GCDFP15-, CDX2-, TTF1-DOG1-, CEA-, CK5-, p40-, D2-40-, Calretinin-, Chromogranin A- |

| | | | | | | |
|---|---|---|---|---|---|---|
| AGN913* | M | 53 | >93 (Alive 08/2020) | Multiple lymph nodes (sub-diaphragmatic) | CUP, adenocarcinoma with apocrine features | CK7+, CK20-, AR+, GCDFP15+, p63-, TTF1-, ER-, PSA-, Racemase- |
| AGN918 | M | 57 | 46 | Multiple lymph nodes (supra- and sub-diaphragmatic), bone, kidney, brain, bone marrow | CUP, poorly differentiated adenocarcinoma | CK7+, CK20-, CEA+, MNF116+, CAM5.2+, p16+, CK5+/-, TTF1+/-, CK6-, CDX2-, p40-, p63-, PSA-, Napsin A-, CD10-, CD30-, CD117-, HMB45-, Actin-, Desmin-, S100-, Vimentin-, CD56-, AFP-Synaptophysin-, Chromogranin A-, Thyroglobulin-, Calcitonin- |
| AGN322 | M | 50 | 18 | Ileo-psoas muscle and multiple sub-diaphragmatic lymph nodes | CUP, poorly differentiated adenocarcinoma | CK7+, CK20+ (focal), AE1/AE3+,CK8+,CK19+, MNF116+, MOC31+, DOG1+,TTF1-, CDX2-, Chromogranin A-, Synaptophysin-, Vimentin-, CD30-, p40-, D2-40- |
| AGN325 | F | 72 | >18 (Alive 01/2021) | Multiple supra-diaphragmatic lymph nodes, pectoralis minor muscle | CUP, poorly differentiated carcinoma with squamous features | CK5+, CK6+, AE1/AE3+, CDH1+, GATA3-, GCDFP15-, ER-, PR-, TTF1-, p63-, Synaptophysin- |
| AGN327 | F | 52 | 5 | Multiple visceral sites (pleura, pericardium and liver), and lymph nodes (mediastinum) | CUP, poorly differentiated adenocarcinoma | CK7+, CK20+ (focal), CK18+, TTF1+ (focal), CDX2- |
| AGN331 | F | 59 | 7 | Multiple cerebral sites | CUP, poorly differentiated squamous carcinoma | CK7+, CK20-, CK5/6 (focal) p40+, p16+, CD10+ EMA+, BCL2+, CD117+, CD56 (focal), Vimentin (focal), ER-, PR-, HER2-, GATA3-, Melan-A-, CDX2-, TTF1-, Napsin A-, CD34-, Desmin-, Actin (MS)-, PAX8-, SOX10-, WT1-, Chromogranin A- |

| Patients with early metastatic cancer of known origin | | | | | | |
|---|---|---|---|---|---|---|
| **Case ID** | **Sex** | **Age at diagnosis (years)** | **OS (months)** | **Metastatic sites at progression** | **Histopathology** | **Immunohistochemical phenotype** |
| mMERK44 | M | 74 | 14 | Multiple lymph nodes (subdiaphragmatic) | Neuroendocrine carcinoma (Merkel cell) | CK7-, CK20+, CD56+, PAX5+, TdT+, NSE+, Synaptophysin+, Chromogranin A+, Neurofilaments+, TTF1- |
| mMEL321* | M | 64 | >18 (Alive 10/2020) | Multiple visceral sites, and mesenteric lymph node | Intestinal melanoma | Melan A+, S100+, Synaptophysin +, MNF116-, CD117- |
| mBRE35 | F | 39 | 47 | Axillary lymph nodes | Breast, poorly differentiated carcinoma | CK7+, CK20-, AE1/AE3+, ER+(focal), HER2+, GCDFP-15+, MOC31+, CDH1+, CDX2-, TTF1-, Napsin A- |
| mBRE40 | F | 63 | 33 | Multiple (supra- and sub-diaphragmatic) bone sites (humerus, vertebrae, sternum and pelvis), liver, axillary lymph nodes | Breast, invasive lobular carcinoma | ER+, PR+, HER2- |
| mBRE45 | F | 96 | 16 | Axillary lymph nodes | Breast, invasive carcinoma | CK7+, CK20-, AE1/AE3+, TTF1+, ER (focal), GATA3+, Calcitonin+, Synaptophysin (focal), HER2- |
| mSKIN49 | M | 72 | 19 | Skin and bilateral lymph nodes of groin | Skin, non-melanocytic adenocarcinoma (squamous and clear cell differentiation) | CK7+, CK20+, AE1/AE3+, CK5+, PSA-, CDX2-, TTF1-, GCDFP15-, LCA-, Vimentin-, Racemase-, Melan-A-, Chromogranin A- |
| mH&N50 | M | 65 | 6 | Neck lymph nodes | Salivary glands Carcinosarcoma | CK14+, CK15+, Vimentin+, S100-, p16-, Calponin- |
| mH&N54 | M | 72 | >38 (Alive 07/2020) | Neck lymph node | Larynx, squamous cell carcinoma | AE1/AE3+, CK5, p40+, p63+, TTF1-, Thyroglobulin- |
| mBRE56 | F | 55 | 33 | Bone, liver | Breast, invasive carcinoma (solid-trabecular pattern) | GCDFP15+, HER2+, GATA3+, TTF1-, ER-, PR-, Thyreoglobulin-, Synaptophysin- |
| mSKIN61 | M | 76 | 4 | Skin-soft tissue, multiple lymph nodes | Skin, adenocarcinoma (Paget disease derived) | CK7+, CK20-, GATA3+, MOC31+, CDX2-, CD117- |
| mBRE923 | F | 48 | 27 | Multiple supra- and sub-diaphragmatic sites (bones, viscera and lymph nodes) | Breast, mixed ductal-lobular invasive carcinoma | CK7+, CK20-, AE1/AE3+, BCA225+ CK19+, ER+, PR+, CDH1+, WT1-, GCDFP15-, HER2 (score 1+), CA125- |
| **Patients with melanoma** | | | | | | |
| MEL2 ** | M | 73 | 18 | Multiple lymph nodes (abdomen and groin), intestine (ileum) | Epithelial Melanoma | Melan-A+ (focal), HMB45+ (focal), S100+ (focal) |
| MEL4** | F | 80 | 23 | Parotid | Epithelial melanoma | Melan-A+, HMB45+, S100+, AE1/AE3- |

*: cases providing fresh tissues or patient-derived xenografts for agnosphere or melanosphere derivation.

**: transcriptome analysis was performed on lymph node metastasis (MEL2) and primary melanoma (MEL4).

AR: Androgen Receptor; CK: Cytokeratin; CUP: Cancer of Unknown Primary origin; ER: Estrogen Receptor; GCDFP-15: Gross Cystic Disease Fluid Protein-15; BCA225: Breast Cancer Antigen 225; CA125: Cancer

Antigen 125; CDX2: Caudal type Homeobox 2; CEA: CarcinoEmbryonic Antigen; EMA: Epithelial Membrane Antigen; HepPar-1: Hepatocyte specific antigen; HMB45: Human Melanoma Black-45; LCA: Leukocyte Common Antigen; LN: Lymph nodes; NSE: Neuron Specific Enolase; OS: overall survival; PR: Progesterone Receptor; PSA: Prostate Specific Antigen; TdT: Terminal deoxynucleotidyl Transferase; TTF1: Thyroid Transcription Factor 1; WT1: Wilms Tumor 1; (+): positive expression; (-): negative expression.

**Supplementary Table 2. Summary of human CUP tissue engraftment in NOD/SCID mice and agnosphere generation**

| Case ID | Original tissue sampling | PDX generation | Agnosphere generation |
|---|---|---|---|
| AGN901 | Lymph node (axilla) surgery | Successful | Successful from PDX |
| AGN906 | Bone (skull)-scalp lesion surgery* | Successful | Successful from human tissue |
| AGN909 | Subcutaneous (chest) lesion biopsy | Successful | Failed from PDX |
| AGN913 | Lymph node (abdominal) surgery | Failed | Failed from human tissue |
| AGN914 | Muscle (gluteus) biopsy | Successful | Successful from PDX |
| AGN43 | Lymph node (axilla) biopsy | Successful | Successful from PDX |
| AGN47 | Lymph node (groin) surgery | Failed | Successful from human tissue |
| AGN67 | Liver lesion biopsy* | Successful | Successful from PDX |

*The tissue sample from which PDX (Patient-Derived Xenograft) and agnospheres were derived was different from the sample used for diagnosis

**Supplementary Table 3. Cancer-associated genes with pathogenic alterations found in CUP human samples and agnospheres**

| Case ID | Gene ID | Gene Type | Variant allele frequency (%) | | Pathogenic FATHMM[a] score |
|---|---|---|---|---|---|
| | | | Human tissue | Agnosphere | |
| 901 | *POLE*[b] | TSG | 12 | 34 | 0.995 |
| 906 | *HRAS* | Oncogene | 16 | 80 | 0.994 |
| | *PIK3CA* | Oncogene | <10 | 100 | 0.995 |
| | *TP53* | TSG | 16 | 98 | 0.874 |
| | *TSHR* | Oncogene | <10 | 60 | 0.920 |
| 43 | *ARID2* | TSG | 39 | 100 | 0.996 |
| | *CREBBP* | TSG | 30 | 100 | * |
| | *CTNNA2* | Oncogene | <10 | 69 | 0.993 |
| | *DAXX* | Oncogene | 27 | 98 | 0.906 |
| | *GATA2* | Oncogene | 19 | 47 | 0.983 |
| | *GATA3* | Oncogene | 16 | 46 | 0.936 |
| | *KEAP1* | TSG | 22 | 100 | 0.989 |
| | *NTRK*[c] | Oncogene | 27 | 59 | 0.996 |
| | *SMARCA4* | TSG | 21 | 99 | 0.991 |
| | *TP53* | TSG | 29 | 100 | * |
| | *UBR5* | TSG | 18 | 49 | 0.992 |
| | *ZHFX3* | TSG | 32 | 99 | 0.99 |
| 67 | *POLE*[b] | TSG | N/A | 50 | 0.993 |
| | *POLQ*[b] | TSG | N/A | 56 | 0.981 |
| 914 | *ABL2* | Oncogene | N/A | 51 | 0.994 |
| | *ARID1B* | TSG | N/A | 100 | 0.933 |
| | *BCL11H* | TSG | N/A | 48 | 0.800 |
| | *CDH11* | TSG | N/A | 44 | 0.993 |
| | *CSMD3* | TSG | N/A | 57 | 0.981 |
| | *FAT1* | TSG | N/A | 49 | 0.945 |
| | *GNA11* | Oncogene | N/A | 97 | 0.956 |
| | *H3F3B* | Oncogene | N/A | 44 | 0.944 |
| | *KRAS* | Oncogene | N/A | 100 | 0.979 |
| | *MYB* | Oncogene | N/A | 100 | 0.933 |
| | *TP53* | TSG | N/A | 100 | * |
| | *ZMYM3* | TSG | N/A | 48 | 0.981 |
| 47 | - | - | - | - | - |

List of oncogenes and tumor suppressor genes (TSG) as annotated by the Network of Cancer Genes 6.0 (http://ncg.kcl.ac.uk/), which display a mutation with a pathogenic FATHMM score >0.7. TSG with FATHMM scores ≤0.7 (*) but with a variant allelic frequency approximating 100% in agnospheres are listed as well. Complete WES analysis of somatic mutations (SNVs and InDels) in CUP tissues and agnospheres were deposited

in the European Gnome-phenome Archive (https://ega-archive.org/) under the accession code EGAD00001006668.

[a]Based on Functional Analysis through Hidden Markov Models (FATHMM) v2.3 (http://fathmm.biocompute.org.uk/).

[b]POLE or POLQ mutation correlates with a hypermutator phenotype (AS901 mutational burden: 379,85 nonsyn. mut./Mb; AS67: 227,06 nonsyn. mut./Mb).

[c]*NTRK* mRNA is not expressed in AS43.

N/A: not available.

## Supplementary Notes

### Composite karyotype of agnospheres, Related to Figure 1

AS906. The analysis showed a highly complex karyotype with a near-diploid modal number and multiple numerical and/or structural aberrations with few marker chromosomes. The composite karyotype assessed by G-bands (Figure 1C) and M-FISH analysis (Figure 1D), including all clonal chromosomal aberrations observed in sixteen metaphases, was:

44~57,X,−X,+2,+der(3)t(3;8)(q12;q12),−4,ins(4;7)(q21;q11q32),+5,der(6)t(6;15)(q23;q23), del(7)(q21q36),−8,der(8)t(8;14)(p23;q11),+der(9)t(9;16)(p24;q11),+der(11)t(11;17)(q13;q21),+der(12)t(11;12)(q21;p13.2),+der(12)t(8;12)(q12;q12)t(8;11)(q24;q13),der(13)t(2;13)(?p13;p11),der(13;19)(q10;q10),−14,14,der(15)t(14;15)(q11;p11)t(13;14)(q14;q32),+dic(16;22)t(16;22)(p13;q13)dup(22)(q11q13),+der(17)t(10;17)(q11.2;p13.3),−18,+20,−22,+1~2mar[cp16]. Numerical and structural alterations involved all chromosomes, except chromosomes 1, 10 and 21.

AS43. A highly complex karyotype with two clones was observed. The main clone (77% of metaphases) was characterized by a near-diploid karyotype, whereas the second one showed a hypo-triploid karyotype (22%). All cells had multiple numerical and/or structural aberrations and few marker chromosomes of unknown origin. The composite karyotype, including all clonal chromosomal aberrations was:

The main clone:

44~57,Y,?der(X)t(X;2)(q13;?),+Y,der(1)?dup(1)(q32q43)?t(1;1)(p3?5;q25),+der(1;6)(q10;q10),+der(1;6)(q10;q10),+der(1;21)(q10;q10),del(2)(q31q3?6),+3,−4,del(4)(q31q35),+del(5)(q12q34),+der(5;13)t(5;13)(p10;q10),add(6)(q11),der(6)t(6;17)(q11;?),−8,der(8)t(3;8)(?;q?), del(9)(p13),−11,+12,−13,der(13)t(8;13)(?;p11),+der(14;19)(q10;p10),-18,-21,+1~3 mar [cp7]

The second clone:

59,XYY,+?der(X)t(X;2)(q13;?),der(1)?dup(1)(q32q43)?t(1;1)(p3?5;q25),der(1;6)(q10;q10), +der(1;6)(q10;q10),+der(1;21)(q10;q10),del(2)(q31q3?6),−4,del(4)(q31q35),del(5)(q12q34) x2,+der(5;13)t(5;13)(p10;q10),−6,add(6)(q11),der(6)t(6;17)(q11;?),−8,der(8)t(3;8)(?;q?),del (9)(p13),−11,−13,−13,der(13)t(8;13)(?;p11),der(14;19)(q10;p10),−17,−18,−19,-21,-22,+1~3mar [cp2].

AGN67. The cytogenetic analysis on twenty metaphases of AGN67 cells showed a highly complex karyotype with a hyper-triploid–modal number. This cell line has multiple numerical and/or structural aberrations and few marker chromosomes.
The composite karyotype assessed by G-bands and M-FISH analysis, including all clonal chromosomal aberrations observed in twenty metaphases, was:
70~77,XX,−X,+der(1;9)(?;q12)del(1)(?),+der(1;17)(q10;q10),+i(2)(q10),+?der(1;2)(?;q10)del(2)(q21q22),−3,der(4)t(4;7)(q35;q35),der(4)dup(4)(q13q32)t(4;7)(q35;q35),+del(6)(p12),+del(6)(q21q26),+del(7)(q22q36),der(9)t(3;9)(q24;q34),+12,i(14)(q10),+15,−17,+19,+20,der(21)t(3;21)(q25;q22),der(22)t(3;22)(q23;q13)x2,+i(22)(q10),+1~3 mar [cp=20].

AGN914. The cytogenetic analysis on twenty metaphases of AGN914 cells showed a highly complex karyotype with a near-diploid–modal number. This cell line has multiple numerical and/or structural aberrations and few marker chromosomes.
The composite karyotype assessed by G-bands and M-FISH analysis, including all clonal chromosomal aberrations observed in twenty metaphases, was:
49~55,X,add(X)(p13),dup(1)(q44q12),der(2)t(2;7)(q21;q22),der(2;11)(q32;q13),−5,+der(7;8)(q10;q10),der(8)t(5;8)(q31;q24),+der(10)?t(10;15)(q24;p12),der(11)t(3;11)(q13;p12),+der(11)t(2;3;11)(?;q22;q13.4),del(12)(p12p13),del(12)(p12p13),+inv(12)(p12q13),−13,der(15)t(?10;15)(p11;p11),add(17)(p13)x2,−18,der(19)t(13;19)(p13;q13),der(20;22)(q10;p10),der(22)t(?5;22)(q21;q13),+1~5 mar [cp20].

N-AS47. The analysis displayed a complex karyotype with three different but related clones, as they shared the majority of chromosomal aberrations.
i) The main clone (53% of metaphases) exhibited a near-triploid karyotype: 60~80,XX,-X,+dup(1)(p31p36),del(2)(q31q35),+3,t(3;9)(q21;q21),+4,+5,+8,−10,+11,+12,−13,+14,−15,+16,+19,+20,+dup(21)(q22q21),+22,+mar[cp7].
ii) The second clone showed a near-diploid karyotype (30%):
44~52,XX,dup(1)(p31p36), del(2)(q31q35),t(3;9)(q21;q21),-10,+19,dup(21)(q22q21)[cp4].
iii) The third was a near-tetraploid clone (10%):
83~86,XX,dup(1)(p31p36)x2,del(2)(q31q35)x2,t(3;9)(q21;q21)x2,−7−10,−10,15,dup(21)(q22q21)x2[cp2].
This agnosphere had mostly multiple numerical aberrations and few structural anomalies, the latter being present in all different clones, with the exception of the marker that was observed only in the near triploid clone.

## Supplementary Methods

### gDNA extraction from tissues

Fresh human CUP tissue specimens were collected, incubated O/N in RNA later (Life Technologies) and quick-frozen at -80°C. Samples were minced and gDNA isolated using Relia Prep™ gDNA Tissue Miniprep System (Promega) according to manufacturer's instructions. Normal gDNA was also derived from micro-dissection of a FFPE normal tissue section and extracted as above (AGN906), or from peripheral blood mononuclear cells (PBMCs) using the Maxwell RSC Whole Blood DNA Kit (Promega) according to manufacturer's instructions. DNA was quantified using Nanodrop ND1000 spectrophotometer (Thermo Fisher Scientific) and Qubit 4 Fluorometer (Thermo Fisher Scientific).

### Whole exome sequencing

Whole Exome Sequencing was performed using 1μg of gDNA, quantified using a Qubit 4 Fluorometer and enriched with the SeqCap EZ MedExome Probes (Roche) targeting the human exome, and then sequenced on NEXTSeq500 (Illumina).

### Sequence alignment and variant annotation

Sequencing reads from each sample were aligned to the human genome (hg38) using Burrows-Wheeler Aligner (BWA, RRID:SCR_010910) mem[1] with default parameters. PCR duplicates were removed using rmdup (SAMtools, RRID:SCR_002105)[2]. Only reads uniquely mapping in the targeted regions were retained for further analysis. Somatic single nucleotide variants (SNVs) and small insertion/deletions (InDels) were identified using Strelka2[3], comparing each tumor with the corresponding normal sample. Somatic SNVs and InDels were further retained if they (*i*) were supported by at least 10 mutated reads in

the tumor; (*ii*) had allele frequency≥5%; (*iii*) were supported by less than one mutated reads in the normal and (*iv*) had a reported Empirical Variant Scoring (EVS) by STRELKA≥15 (RRID:SCR_005109). Finally, ANNOVAR, RRID:SCR_012821[4] was used to annotate non-silent (nonsynonymous, stopgain, stoploss, frameshift, non-frameshift and splicing modifications) somatic mutations in each tumor.

**Cell viability in response to growth factors and inhibitors**

Agnospheres or melanospheres were dissociated and seeded in culture medium in 96-well microtiters at the concentration of 200 cells/100μl in the case of AS901, AS906, AS43, AS67 and mMS321, 500 cells/100μl for AS914 and 1000 cells/100μl for N-AS47. To assess growth factor response, EGF, bFGF, HGF, PDGF, IGF2 (20ng/ml, Peprotech) and NRG1 (10 ng/ml, Peprotech) were added immediately after seeding, and cell viability was analyzed at day 4. To assess response to inhibitors, all treatments started immediately after seeding: lapatinib (0.5 μM, Carbosynth) or selumetinib (0.5 μM, Selleckchem) were added daily, afatinib (0.5 μM, Carbosynth) every other day, and trametinib (50 nM, Selleckchem) every 5 days. ATP consumption was measured at day 4, 9, 10 or 14 after cell seeding with Cell Titer Glo (Promega) according to manufacturer' instructions using a GloMax 96 Microplate Luminometer (Promega). In each experiment the average of relative luminescence values (n≥6 technical replicates) of treated samples was normalized vs. untreated controls and fold changes were reported (n≥3, mean±SEM, *p< 0.05, **p<0.01, ns: not significant, ANOVA, Bonferroni multicomparison test).

**Metastatic emboli analysis**

From mice transplanted *subcutis* with AS43 and treated, after tumor establishment, with trametinib or control until the experimental endpoint (tumor volume = 1600 $mm^3$) lungs and heart were explanted and formalin-fixed paraffin-embedded. Complete organ serial

sectioning (5μm thickness) followed by H&E staining was performed, and whole images acquired with D SIGHT 2.0 scanner (Menarini diagnostics). The total number of metastatic emboli was counted in each lung pair. In each embolus, the total number of cells was counted across sections spaced 20μm (1 out of 4 serial sections) to avoid duplicating counts of the same cells. Statistical significance was assessed by Wilcoxon test, 1 tail, p=0,0497.

## Reagents

| Antibody (cat Number) | Concentrations | Company | RRID |
|---|---|---|---|
| AKT (9272) | 1:1000 WB | Cell Signaling Technologies | AB_329827 |
| BMI1, clone D20B, (6964) | 1:100 IF; 1:1000 WB | Cell Signaling Technologies | AB_10828713 |
| c-MYC, clone D84C12 (5605) | 1:50 IF; 1:1000 WB | Cell Signaling Technologies | AB_1903938 |
| CD44, clone 8E (5640) | 1:100 IF; 1:1000 WB | Cell Signaling Technologies | AB_10547133 |
| Cleaved-PARP ASP214 (9541) | 1:1000 WB | Cell Signaling Technologies | AB_331426 |
| EGF Receptor, clone D38B1( 4267) | 1:100 IF; 1:1000 WB | Cell Signaling Technologies | AB_2246311 |
| EpCAM (14452) | 1:1000 WB | Cell Signaling Technologies | AB_2736866 |
| EZH2 (5246) | 1:100 IF; 1:1000 WB | Cell Signaling Technologies | AB_10694683 |
| HER3, clone1B2 (4754) | 1:1000 WB | Cell Signaling Technologies | AB_10691324 |
| IGF1Rb (3027) | 1:1000 WB | Cell Signaling Technologies | AB_2122378 |
| N-MYC, clone D1VA2 (84406) | 1:1000 WB | Cell Signaling Technologies | AB_2800038 |
| NANOG (4903) | 1:1000 WB | Cell Signaling Technologies | AB_10559205 |
| p-AKT Ser473, clone 736E16 (3787) | 1:1000 WB | Cell Signaling Technologies | AB_331170 |
| p-EGFR Y1068, clone 15A2 (2234) | 1:100 IF; 1:1000 WWB | Cell Signaling Technologies | AB_331701 |
| p-HER3 Y1289 (4791) | 1:1000 WB | Cell Signaling Technologies | AB_2099709 |
| p-p38 T180/Y182 (9215) | 1:1000 WB | Cell Signaling Technologies | AB_331762 |
| p27 (3688) | 1:1000 WB | Cell Signaling Technologies | AB_2077836 |
| p38 (9212) | 1:1000 WB | Cell Signaling Technologies | AB_330713 |
| pHER2 Y1248 (2247) | 1:1000 WB | Cell Signaling Technologies | AB_331725 |
| pMEK1/2 S217/221 (9154) | 1:1000 WB | Cell Signaling | AB_2138017 |
| pSTAT1 Y701 (9167) | 1:1000 WB | Cell Signaling Technologies | AB_561284 |
| pSTAT3 Y705 (9131) | 1:1000 WB | Cell Signaling Technologies | AB_331586 |
| SOX2 (3579) | 1:1000 WB | Cell Signaling Technologies | AB_2195767 |
| STAT1, clone D4Y6Z (14995) | 1:1000 WB | Cell Signaling Technologies | AB_271628 |
| Survivin | 1:1000 WB | Cell Signaling Technologies | AB_2063948 |
| YAP/TAZ, clone D24E4 (8418) | 1:1000 WB | Cell Signaling Technologies | AB_10950494 |
| E-Cadherin (610182) | 1:1000 WB | BD | AB_397581 |
| MET (C-12) | 1:1000 WB | Santa Cruz | AB_631940 |
| NEU (CB11) | 1:1000 WB | Santa Cruz Biotechnology | AB_630029 |
| SLUG, clone A-7 (sc-166476) | 1:1000 WB | Santa Cruz Biotechnology | AB_2191897 |
| TUBB2 clone 1A9 (sc-134230) | 1:1000 WB | Santa Cruz Biotechnology | AB_2272485 |
| KLF4, clone 1E6 (MABC631) | 1:100 IF | Millipore | AB_2858269 |

| | | | |
|---|---|---|---|
| OCT4, clone 10H11.2 (MAB4401) | 1:1000 WB | Millipore | AB 2167852 |
| Vimentin (CBl202) | 1:100 IF; 1:1000 WB | Millipore | AB 93387 |
| ZEB1 (HPA027524) | 1:1000 WB | SIGMA | AB 1844977 |
| pIGF1R Y1161 ABP-0367 | 1:1000 WB | Immunological science | AB 2861138 |
| pMET1349 (ABP11206) | 1:1000 WB | Immunological science | AB 2858270 |
| pERK1/2 T202/Y204 (MAB94122) | 1:1000 WB | Immunological science | AB 2858271 |
| phospho-pRB S807 (ABP-0648) | 1:1000 WB | Immunological science | AB 2861137 |
| SOX2 (AF2018) | 1:30 IF | R&D | AB 47762 |
| STAT3 (MAB1799) | 1:1000 WB | R&D | AB 2198753 |
| YAP (13584-1-AP) | 1:2000 WB | Proteintech | AB 2218915 |
| BCA225, clone CU18 (ab3360) | 1:50 IHC | Abcam | AB 303739 |
| CD-56 (NCL-CD56-1B6) | 1:80 IHC | Leica | AB 563906 |
| Chromogranin A (PA0430) | ready to use,IHC | Leica | AB 10554754 |
| Vimentin (NCL-LVIM-572) | 1:400 IHC | Leica | AB 10555996 |
| Cytokeratin 5 (TA800728) | 1:250 IHC | OriGene Technologies | AB 2625644 |
| Cyclin D1 | 1:500 WB | OriGene Technologies | AB 2259619 |
| p-40 (ACI 3066 C) | 1:100 IHC | Biocare Medical | AB 2858274 |
| CDX2 (IR080) | ready to use,IHC | Agilent | AB 2858275 |
| Cytokeratin AE1/AE3 (M3515) | 1:100 IHC | Agilent | AB 2132885 |
| Cytokeratin MNF116 (M0821) | 1:100 IHC | Agilent | AB 2858276, |
| Cytokeratin 7 (M7018) | 1:80 IHC | Agilent | AB 2134589 |
| Cytokeratin 18 (M7010) | 1:80 IHC | Agilent | AB 2133299 |
| Cytokeratin 20 (M7019) | 1:60 IHC | Agilent | AB 2133718 |
| Synaptophysin (M7315) | 1:80 IHC | Agilent | AB 2687942 |
| Cyclin D1, clone SP4 | 1:100 IHC | ThermoFisher Scientific | AB 149912 |
| Alexa Fluor 647 donkey anti mouse Ig (H+L) (A31571) | 1:750 IF | Jackson Lab | AB 162542 |
| Alexa Fluor 488 donkey anti rabbit Ig H+L) (A21206) | 1:750 IF | Jackson Lab | AB 2535792 |
| Alexa Fluor 555 donkey anti goat Ig (H+L) (A21432) | 1:5000 IF | Jackson Lab | AB 2535853 |
| Peroxidase-conigated AffiPure Goat Anti-Mouse IgG (H L) | 1:20000 WB | Jackson Lab | AB 10015289 |
| Peroxidase-conigated AffiPure Goat Anti-Rabbit IgG (H L) | 1:20000 WB | Jackson Lab | AB 2313567 |
| Peroxidase-conigated AffiPure Rabbit Anti-Goat IgG (H L) | 1:20000 WB | Jackson Lab | AB 2339400 |

| Probe | Assay ID | Company |
|---|---|---|
| EGF | Hs01099999_m1 | ThermoFisher Scientific |
| EREG | Hs00914313_m1 | ThermoFisher Scientific |
| FGF2 | Hs00960934_m1 | ThermoFisher Scientific |
| HBEGF | Hs00181813_m1 | ThermoFisher Scientific |
| HGF | Hs00300159_m1 | ThermoFisher Scientific |
| IGF1 | Hs01547656_m1 | ThermoFisher Scientific |
| IGF2 | Hs04188276_m1 | ThermoFisher Scientific |
| NRG1 | Hs00247620_m1 | ThermoFisher Scientific |
| NRG2 | Hs00171706_m1 | ThermoFisher Scientific |
| PDGFA | Hs00236997_m1 | ThermoFisher Scientific |
| PDGFB | Hs00234042_m1 | ThermoFisher Scientific |
| TGFA | Hs00608187_m1 | ThermoFisher Scientific |
| AREG | Hs00950669_m1 | ThermoFisher Scientific |
| HPRT1 | Hs01003267_m1 | ThermoFisher Scientific |
| EGFR | Hs01076078_m1 | ThermoFisher Scientific |
| ERBB2 | Hs01001580_m1 | ThermoFisher Scientific |
| ERBB3 | Hs00176538_m1 | ThermoFisher Scientific |
| MET | Hs01565584_m1 | ThermoFisher Scientific |
| IGF1R | Hs00609566_m1 | ThermoFisher Scientific |
| PDGFRA | Hs00183486_m1 | ThermoFisher Scientific |
| PDGFRB | HS00387364_m1 | ThermoFisher Scientific |
| GREB1 | Hs01738470_cn | ThermoFisher Scientific |
| PTEN | Hs02599450_cn | ThermoFisher Scientific |
| TP53 | Hs06423639_cn | ThermoFisher Scientific |
| c-MYC | Hs00834648_cn | ThermoFisher Scientific |
| N-MYC | Hs00201049_cn | ThermoFisher Scientific |

## Supplementary References


1 Li, H. & Durbin, R. Fast and accurate long-read alignment with Burrows-Wheeler transform. *Bioinformatics* **26**, 589-595 (2010).

2 Li, H. *et al.* The Sequence Alignment/Map format and SAMtools. *Bioinformatics* **25**, 2078-2079 (2009).

3 Kim, S. *et al.* Strelka2: fast and accurate calling of germline and somatic variants. *Nat Methods* **15**, 591-594 (2018).

4 Wang, K., Li, M. & Hakonarson, H. ANNOVAR: functional annotation of genetic variants from high-throughput sequencing data. *Nucleic Acids Res* **38**, e164 (2010).
