# Supplementary material for: Cancer of unknown primary stem-like cells model multi-organ metastasis and unveil liability to MEK inhibition
Source: Nat Commun. 2021 May 3;12:2498. doi: 10.1038/s41467-021-22643-w (PMC8093243; doi:10.1038/s41467-021-22643-w)
Supplement: Supplementary file 3 — Description of Additional Supplementary Files [file 41467_2021_22643_MOESM3_ESM.docx]

Description of Additional Supplementary Files

**Title:** Supplementary Data 1

**Description:** RNAseq data, Embryonic Stem cell (ES) Signature and trametinib signature of tumorspheres and patient tissues. Sheet 1: Gene set related to the Embryonic Stem cell (ES) signature from Ben-Porath et al., Nat. Genet. 40, 499-507 (2008). For each gene we reported its (A) official Gene Symbol; (B) Ensembl id; and (C) the name of the gene set. Sheet 2: Gene set enrichment analysis (GSEA) of ES related pathways in hESC, agnospheres, tumorspheres, and CUP tissues. For each tested gene set we reported: (A) name; (B) p-value; (C) corrected p-value; (D) enrichment score; (E) normalized enrichment score; and (F) the sample on which GSEA was performed. Sheet 3: GSEA of ES related pathways in breast cancer tissues. For each tested gene set we reported: (A) name; (B) p-value; (C) corrected p-value; (D) Enrichment score; (E) normalized enrichment score; and (F) the sample on which GSEA was performed. Sheet 4: Trametinib signature. For each gene it is reported: (A) Ensembl id; (B) official Gene Symbol; and (C) the description. Sheet 5: Trametinib signature enrichment analysis. For each tested gene set it is reported: (A) name; (B) p-value; (C) corrected p-value; (D) enrichment score; (E) normalized enrichment score; and (F) the sample on which GSEA was performed Related to Figures 2 and 5 and Supplementary Figure 4.
