# Supplementary material for: Cancer of unknown primary stem-like cells model multi-organ metastasis and unveil liability to MEK inhibition
Source: Nat Commun. 2021 May 3;12:2498. doi: 10.1038/s41467-021-22643-w (PMC8093243; doi:10.1038/s41467-021-22643-w)
Supplement: Supplementary file 5 — Reporting Summary [file 41467_2021_22643_MOESM5_ESM.pdf]

nature research

Corresponding author(s): Carla Boccaccio

Last updated by author(s): Feb 25, 2021

Reporting Summary

Nature Research wishes to improve the reproducibility of the work that we publish. This form provides structure for consistency and transparency in reporting. For further information on Nature Research policies, see our Editorial Policies and the Editorial Policy Checklist.

Statistics

For all statistical analyses, confirm that the following items are present in the figure legend, table legend, main text, or Methods section.

n/a	Confirmed	
☐	☒	The exact sample size (*n*) for each experimental group/condition, given as a discrete number and unit of measurement
☐	☒	A statement on whether measurements were taken from distinct samples or whether the same sample was measured repeatedly
☐	☒	The statistical test(s) used AND whether they are one- or two-sided<br>*Only common tests should be described solely by name; describe more complex techniques in the Methods section.*
☒	☐	A description of all covariates tested
☒	☐	A description of any assumptions or corrections, such as tests of normality and adjustment for multiple comparisons
☐	☒	A full description of the statistical parameters including central tendency (e.g. means) or other basic estimates (e.g. regression coefficient) AND variation (e.g. standard deviation) or associated estimates of uncertainty (e.g. confidence intervals)
☐	☒	For null hypothesis testing, the test statistic (e.g. *F*, *t*, *r*) with confidence intervals, effect sizes, degrees of freedom and *P* value noted<br>*Give P values as exact values whenever suitable.*
☒	☐	For Bayesian analysis, information on the choice of priors and Markov chain Monte Carlo settings
☒	☐	For hierarchical and complex designs, identification of the appropriate level for tests and full reporting of outcomes
☒	☐	Estimates of effect sizes (e.g. Cohen's *d*, Pearson's *r*), indicating how they were calculated

Our web collection on statistics for biologists contains articles on many of the points above.

Software and code

Policy information about availability of computer code

Data collection	Image Lab Touch 2.4 software, NIS Elements Imaging software, CytoVision software (Leica Biosystems), LASV4.2 software, IVIS imaging software, GloMax®-96 Software.
Data analysis	For 3'UTR-Seq dataset alignment and expression quantification STAR 2.6a, htseq-count 0.9.1 software were used. Downstream analysis of 3'UTR-Seq was performed in R statistical environment 3.6 by using fgsea R package v1.16, ggplot2 R package v3.3.3 and kernelab R package v0.9. Statistical analyses were performed using commercial software Graphpad PRISM v8. For whole exome sequencing the following software were used: bbmap v37.95, bwa v0.7.17, samtools v1.3, strelka v2.9.2, manta v1.5 and annovar v2019Oct2. Metasytems ISIS software was used for cytogenetic analysis; ELDA was used for limiting dilution assay analyis and Fiji-ImageJ was used for morphometric analyses.

For manuscripts utilizing custom algorithms or software that are central to the research but not yet described in published literature, software must be made available to editors and reviewers. We strongly encourage code deposition in a community repository (e.g. GitHub). See the Nature Research guidelines for submitting code & software for further information.

Data

Policy information about availability of data

All manuscripts must include a data availability statement. This statement should provide the following information, where applicable:

- Accession codes, unique identifiers, or web links for publicly available datasets
- A list of figures that have associated raw data
- A description of any restrictions on data availability

Complete datasets related to WES analysis of somatic mutations, referring to Supplementary Table 3, were deposited in the European Genome-phenome Archive (EGA), under the accession code EGAS00001004868 (https://ega-archive.org/). Raw data relative to 3'UTR-Seq, referring to Fig. 2d, Fig. 5g-l and Supplementary Fig. 1d-e were uploaded in the Gene Expression Omnibus (GEO) repository, under the accession code GSE167473 (https://www.ncbi.nlm.nih.gov/geo/query/acc.cgi?

acc=GSE167473). Source data are provided in the Source Data file. Public datasets from the following databases are available at these web links: Cancer Cell Line Encyclopedia: https://portals.broadinstitute.org/ccle; Genomics of Drug Sensitivity in Cancer: https://www.cancerrxgene.org/.

Field-specific reporting

Please select the one below that is the best fit for your research. If you are not sure, read the appropriate sections before making your selection.

- [x] Life sciences
- [ ] Behavioural & social sciences
- [ ] Ecological, evolutionary & environmental sciences

For a reference copy of the document with all sections, see nature.com/documents/nr-reporting-summary-flat.pdf

Life sciences study design

All studies must disclose on these points even when the disclosure is negative.

Sample size	To identify the significant therapeutic responses with a statistical power of 90%, we calculated the mice sample size by considering: (i) volume variations of ±15% as random; (ii) volume variation of at least 20% as a therapeutic effect; (iii) a statistical significance of p = 0.05. Accordingly, at least 6 mice/group were used.
Data exclusions	For in vivo experiments, mice which prematurely died in the absence of subcutaneus tumors were excluded from counts.
Replication	All in vitro experiments were repeated at least 3 times with different set of cells, unless otherwise indicated in figure legends. In cell viability and LDA experiments a minimum of 6 technical replicates were performed in each condition. For in vivo assessment of metastases a minimum of 13 mice/agnosphere were used, for tumor growth and survival 6 mice/treatment group were used, for in vivo LDA the number of mice/condition has been reported in Fig. 3c and Supplementary Fig.3c. All experimental findings were reliably reproduced and attempts at replication were successful.
Randomization	For in vivo experiments mice were subcutaneously injected with 50.000 cells and once tumors were established and reached 175-200 mm3, mice were randomized in 2 groups using the LAS software (Baralis et al, 2012). For in vitro studies, samples were randomly allocated to experimental groups.
Blinding	For the in vivo therapeutic response the investigator was not blinded, because the same person who treated mice was also responsible for tumor measurements. For the assessment of metastases in vivo, more than 1 investigator contributed to generate the data (in vivo and ex vivo imaging, IHC analysis).

Reporting for specific materials, systems and methods

We require information from authors about some types of materials, experimental systems and methods used in many studies. Here, indicate whether each material, system or method listed is relevant to your study. If you are not sure if a list item applies to your research, read the appropriate section before selecting a response.

Materials & experimental systems

n/a	Involved in the study	
[ ]	[x]	Antibodies
[x]	[ ]	Eukaryotic cell lines
[x]	[ ]	Palaeontology and archaeology
[ ]	[x]	Animals and other organisms
[ ]	[x]	Human research participants
[x]	[ ]	Clinical data
[x]	[ ]	Dual use research of concern

Methods

n/a	Involved in the study	
[x]	[ ]	ChIP-seq
[x]	[ ]	Flow cytometry
[x]	[ ]	MRI-based neuroimaging

Antibodies

Antibodies used	Each antibody (n=63) used was identified by clone, company, catalogue number, concentration and RRID code, as detailed in Supplementary Information (Methods section, Reagents paragraph).
Validation	All 63 antibodies used were commercially available and were validated by the manufacturers for human specificity and for the applications described in the study, as reported in the datasheets.

Animals and other organisms

Policy information about studies involving animals; ARRIVE guidelines recommended for reporting animal research

Laboratory animals	NOD.CB17-Prkdcscid/NcrCr mice (NOD/SCID), (RRID:IMSR_CRL:394, Charles River Laboratories), 5- to 6-week-old male were used for all in vivo studies. Mice were housed at a maximum of 6 per cage with a 14-hour light/10-hour dark cycle, in a conventional animal

	facility with an ambient temperature and humidity of 20-26°C and 40%-60%, respectively, with food and water ad libitum.
Wild animals	No wild animals were used in the study.
Field-collected samples	No field collected samples wre used in the study.
Ethics oversight	All animal procedures were performed according to the institutional guidelines and approved by the Italian Ministry of Health.

Note that full information on the approval of the study protocol must also be provided in the manuscript.

Human research participants

Policy information about studies involving human research participants

Population characteristics	Population charasteristics have been reported in Supplementary Table 1.
Recruitment	Patients with a suspected cancer of unknown primary were enrolled in an approved prospective observational trial where the diagnosis was attaind through a rigorous 'ad excludendum' diagnostic protocol in accordance with European Society of Medical Oncology (ESMO 2018) guidelines. This procedure should minimize the risk of patient selection bias with respect to the diagnosis of CUP.
Ethics oversight	Patients were recruited at the Candiolo Cancer Institute, FPO-IRCCS, according to the ethical requirements of the institutional Review Board on human experimentation. The Review Board approved the observational trial (Study Protocol N. 010-IRCC-10IIS-15, and following updates; last update: v2.0-16.10.2018).

Note that full information on the approval of the study protocol must also be provided in the manuscript.
